# Hydrogen Bonds Stabilize Chloroselenite Anions: Crystal Structure of a New Salt and Donor-Acceptor Bonding to SeO_2_

**DOI:** 10.3390/molecules28227489

**Published:** 2023-11-08

**Authors:** René T. Boeré

**Affiliations:** 1 Department of Chemistry and Biochemistry, University of Lethbridge, Lethbridge, AB T1K 3M4, Canada; boere@uleth.ca; 2 Canadian Centre for Research in Applied Fluorine Technologies (C-CRAFT), University of Lethbridge, Lethbridge, AB T1K 3M4, Canada

**Keywords:** halochalcogenite(IV) ion, crystallography, H-bonding, chalcogen bonding, π-holes, DFT-computed vibrational spectra

## Abstract

The single-crystal X-ray diffraction structure characterizing a new 4-methylbenzamidinium salt of chloroselenite [C_8_H_11_N_2_][ClSeO_2_] is reported. This is only the second crystal structure report on a ClSeO_2_^−^ salt. The structure contains an extended planar hydrogen bond net, including a double interaction with both O atoms of the anion (an $R_{2}^{2}\left(8\right)$ ring in Etter notation). The anion has the shortest Se–Cl distances on record for any chloroselenite ion, 2.3202(9) Å. However, the two Se–O distances are distinct at 1.629(2) and 1.645(2) Å, attributed to weak anion–anion bridging involving the oxygen with the longer bond. DFT computations at the RB3PW91-D3/aug-CC-pVTZ level of theory reproduce the short Se–Cl distance in a gas-phase optimized ion pair, but free optimization of ClSeO_2_^−^ leads to an elongation of this bond. A good match to a known value for [Me_4_N][ClSeO_2_] is found, which fits to the Raman spectroscopic evidence for this long-known salt and to data measured on solutions of the anion in CH_3_CN. The assignment of the experimental Raman spectrum was corrected by means of the DFT-computed vibrational spectrum, confirming the strong mixing of the symmetry coordinate of the Se–Cl stretch with both ν_2_ and ν_4_ modes.

## 1. Introduction

**Scheme 1:**
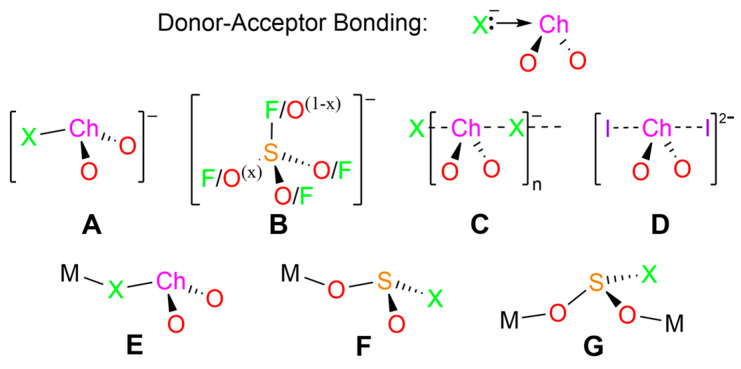
Donor–acceptor bonding in halochalcogenite(IV) ions and schematics of the structure types **A**–**G** known from SC-XRD data; Ch = chalcogen (S, Se, Te); X = halide (F, Cl, Br, I).

The chloroselenite ion, ClSeO_2_^−^, is found in several salts crystallized from the addition of chloride ions to selenium(IV)oxide in non-aqueous solutions [1]. Conceptually, chloroselenite ions are donor–acceptor adducts between the halogen and the chalcogen dioxide (Scheme 1) and they were first identified by direct addition reactions in aprotic solvents. The Cambridge Structural Database (release 2023.2.0) [2] currently lists just one crystal structure [Me_4_N][ClSeO_2_], assigned the CSD Refcode BIRHOZ, but to our knowledge, no atom coordinates are available for this structure in publications or databases. This structure is most consistent with type **A** in Scheme 1, described as having ‘monomeric pyramidal anions’ with *d*(Se–Cl) = 2.453(1); *d*(Se–O) = 1.632(2) Å [3,4,5,6]. The Se–Cl bond length, almost 14% longer than the sums of the covalent radii, is consistent with a weak donor–acceptor bond (for further details on this structure, see the Supplementary Materials). A 2,2′-bipyridium salt [C_10_H_9_N_2_][ClSeO_2_] is indexed in Chemical Abstracts (Registry number [27380-14-9]), but its crystal structure is apparently only available in an unpublished thesis [7]. The anion has been identified in various solvents via liquid-phase Raman spectroscopy as well as in isolated Me_4_N^+^ and Ph_4_As^+^ salts, largely through the systematic work of the Canadian chemist John Milne (1934–2022) [1]. The ^77^Se NMR spectrum of solid [Me_4_N][ClSeO_2_] was noteworthy for having anisotropic shielding of >1000 ppm [8]. The dominance of 1:1 adducts between Cl^−^ ions and SeO_2_ in several aprotic solvents was originally established using UV-vis absorption spectroscopy [9], but the non-existence of the parent acid ClSe(O)OH in aqueous solutions of SeO_2_ in either dilute or concentrated HCl has been attested [10,11]. Similarly, there is no evidence for stable salts of simple metal cations M[ClSeO_2_] despite reported attempts to obtain these [12].

The nature and scope of weak chemical bonds has become a major focus of research in recent years [13]. Chloroselenites are currently of interest in relation to the speciation and extraction of Se(IV) and Se(VI) for environmental concerns [14]. The electron affinities of SeO*_n_* clusters have been evaluated for similar motivations [15] and fundamental spectroscopy on SeO_2_ rotational lines remain of interest for astronomic detection [16,17,18]. Selenium compounds are central to the surge in interest in chalcogen bonding [19,20,21,22,23]. Importantly, SeO_2_ itself has also been identified as having electrostatic ‘π-holes’ [24], which may be of direct relevance to the formation of the halogen adducts [XChO_2_]^−^ (X = halogen; Ch = chalcogen). New attention to the higher oxochloroselenates, after a long hiatus, is bearing fruit with the report of an inclusion compound of Cl_2_ in a tetramethylamino salt of [Se_2_O_2_Cl_7_]^3−^ [25], which harkens back to a much older structure [26]. The thermochemical properties of the fluoroselenite ion have been assessed in a large prospective study [27]. Less is known about the heavier halide adducts of SeO_2_, and halotellurites remain rare. However, there is active research on all the halosulfites due to the recognition of the importance of SO_2_ as a Lewis acid relative to is capability as a solvent medium and environmental hazard, with important structural [28,29,30] and computational studies [31]. The application of modern speciation, structural, and theoretical techniques makes the study of weakly bonded adducts, such as those encountered amongst the halochalcogenites [XChO_2_]^−^, more feasible than ever before.

Despite their simple constitution, there is a dearth of confirmed structural evidence on [XChO_2_]^−^ salts, all of which so far are from single-crystal X-ray diffraction (SC-XRD) data. Since the literature is very scattered, the current state of knowledge is briefly reviewed. FSO_2_^−^ salts are the best represented, with nine known structures, consistent with the accepted wisdom that this is the most stable member of its class (the order of X–Ch bond strength is believed to be F > Cl < Br < I, but this may be Ch-dependent) [32,33]. A phosphonium ylid salt of FSO_2_^−^ was the first reported structure (CSD refcode: LIHWAA) but it suffers from serious F/O positional disorder [34]. Another, aprotic, imidazolium salt (CSD refcode: TOSXEE), also displays a disordered anion [35]. This theme continues for the metal salts K[FSO_2_], Rb[FSO_2_] [36] and the α- and β- polymorphs of Cs[SO_2_F] [30]. The recognition of O/F positional disorder led to a determined but only partially successful attempt to overcome the phenomenon with the preparation and structures of [(Me_2_N)_3_S]^+^, [(Me_2_N)_3_SO]^+^ and [(Me_4_N)_4_N]^+^ salts (CSD refcodes: ADEJOI, ADEJUO and ADEKAN, respectively) [29]. The structure type that best describes all the FSO_2_^−^ salts is **B** in Scheme 1, due to the positional exchange of the very similar-sized O and F, and further positional disorder that often lends a pseudo-tetrahedral appearance to the anion in these structures (the value of *x* can range from 0 to 0.5).

Two chlorosulfite, ClSO_2_^−^, ion structures are in the CSD. The oldest structure (refcodes: POMBEY [37]) contains an isolated ion of type **A** with an S–Cl bond that is 23% longer than the sums of the covalent radi. Much more recently, structure XEGCAQ [38] was reported, wherein one oxygen coordinates to Li^+^, lending it structure type **F**. Fascinatingly, there are also two structures in the database, KIGZEF [39] and LAQYOR [40], which contain infinite chains in which the chloride ions bridge the SO_2_ molecules more or less equally, i.e., structure type **C**. Similar, though far more symmetrical, chain structures of type **C** are displayed by [Et_4_N][BrSO_2_], LAYTUC [28] and by [Me_4_N][BrSeO_2_], BIRHUF [3,4,5].

The remainder are ISO_2_^−^ salts, and these are the most structurally diverse of all. The [Ph_3_PBz]^+^ salt BZTPPI [41] and WUKQUR [42] are both of type **A** (with S–I lengths 38% and 24% longer than sums of the covalent radii), although WUKQUR has a positional disorder that reduces the accuracy of the derived parameters. Structures MPICSO [43] and MPTPIS [44] are of type **E** in which SO_2_ forms an adduct to a metal-coordinated iodide ion. The iodide adduct in WUQMED is another with oxygen coordinated to a metal, type **F**, whereas WUQLUS is a variant on this theme with both oxygen atoms of the SO_2_ attached to separate metals, type **G** [42]. Finally, in DOTXOA, there is a discrete I_2_SO_2_^2−^ ion of structure type **D** [45].

In summary, there are still relatively few known structures for this class, many of which are problematic, and there is a very wide structural diversity, particularly with regards to the X–Ch bond lengths. This situation is consistent with what may be anticipated from weak donor–acceptor bonding. Thus, when we happened on a good quality structure containing a chloroselenite ion, quite by accident, we immediately recognized its importance. Our structure is the first of its kind where paradigmatic hydrogen bonding to the ClSeO_2_^−^ ion has been established, as well as having the shortest Cl–Se bond for chloroselenites. Herein, we provide a full report on this interesting structure and analyze the anion geometry and bonding through extensive B3PW91/aug-cc-pVTZ density functional theory (DFT) computations.

## 2. Results

### 2.1. Formation of the Salt from a Hydrolysis Reaction

**Scheme 2:**
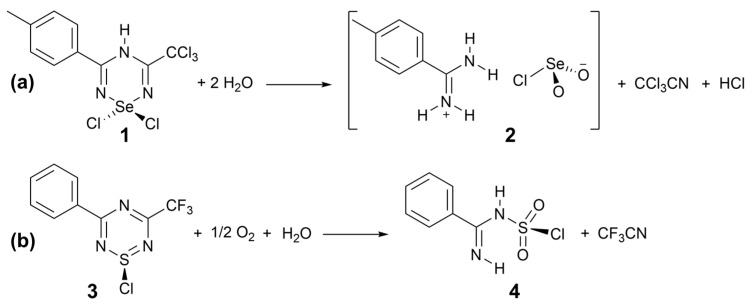
Plausible reactions leading to (**a**) chloroselenite salt **2** from the dichloroselenatriazine **1** or (**b**) sulfamyl chloride **4** from chlorthiatriazine **3**.

In our work on heterocyclic thiazyls and selenazyls, we have explored the synthesis of 1,2,4,6-thiatriazinyl radicals via the reduction of 1-chloro-1,2,4,6-thiatriazines [46,47] and are now extending this work to the selenium analogues. Attempts to recrystallize extremely insoluble selenatriazine **1** for purification used boiling acetonitrile (Scheme 2). **1** is analogous to 1,1-dichloro-3,5-diphenyl-4H-1,2,4,6-selenatriazine, which displays H-bonding in its crystal structure (CSD refcode: DUVDUT), likely the origin of the insolubility [48]. Colorless crystals of **2** were the only identifiable product of the reaction mixture, which has been unambiguously characterized by SC-XRD. This interesting reaction, producing at once the rare chloroselenite ion and the 4-methylbenzamidinium cation, may be contrasted with our earlier observation that hydrolysis of 1-chloro-3-phenyl-5-trifluoromethyl-1λ^4^,2,4,6-thiatriazine **3** [46] in the presence of air forms the covalent (imino(phenyl)methyl)sulfamyl(VI) chloride **4**. Thus, in the formation of **2**, selenium demonstrates its well-known resistance to adopting the highest group oxidation state, a characteristic that is usually attributed to the Scandide contraction [49]. The two products provide an appealing contrast, yet both are remarkable for retaining a Ch–Cl bond and are evidently stabilized by similar H-bonding networks (see below). The full structural and computational characterization of salt **2** follows. For a depiction of the interesting molecular structure of **4**, including its H-bonds, see Appendix B.

### 2.2. Crystallographic Characterization

**Figure 1:**
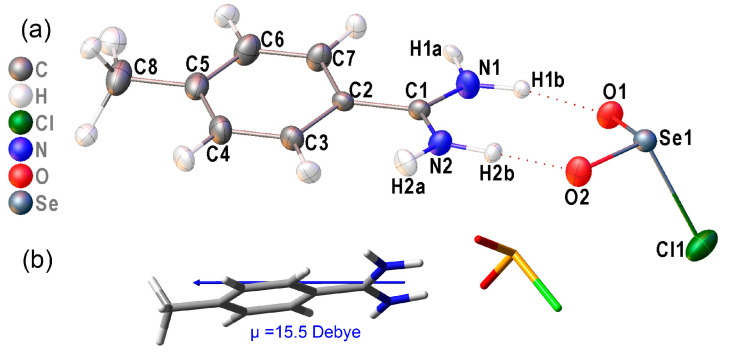
(**a**) Displacement ellipsoids plots (50% probability) for the molecular structures of **2** as found in the crystal lattice. The second component of the CH_3_ rotational disorder model is omitted. H-bonds are shown with red dotted lines. (**b**) Tubes plot, showing the DFT-computed dipole moment (blue vector; IUPAC convention) of an isolated ion pair in **2** from an RB3PW91-D3/aug-CC-pVTZ calculation.

From the SC-XRD data, a structure model for **2** with restrained full refinement of the hydrogen atom positions and displacements was developed by applying Hirsfeld atom refinement (HAR). This method employs custom aspherical atomic scattering factors, computed on the fly by density functional theory (DFT) methods, under the control of the NoSpherA2 package [50] within Olex2 release 1.5 [51]. This approach is particularly useful when H-bonding is present, as it avoids having to normalize E–H bond lengths as otherwise required with XRD structures [52]. Full details of the refinement strategy are provided in the Experimental section. The ion pair structure in **2** is shown in Figure 1, the extended H-bond network and inter-anion contacts are shown later. The derived interatomic parameters have been placed in Table 1, while the H-bond and short contact data are presented in Table 2.

**Figure 2:**
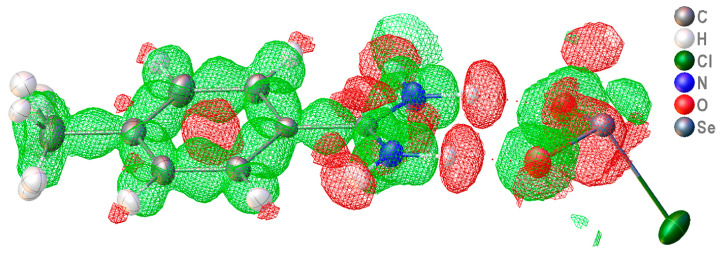
Deformation density map from the HAR/NoSpherA2 refinement of **2**. T If you are certain that the sharing Convention to which you subscribe permits this alteration, you are free to add the reference to the end of the reference list. The lone-pair density above and right of the Se atom is small but distinguishable.

The ion pair structure obtained for **2** (Figure 1a) consists of ClSeO_2_^−^ ions that are doubly hydrogen-bonded to the toluamidinium H atoms H1b and H2b. There is, to date, only one set of comparison data in the literature, the aforementioned BIRHOZ structure of [Me_4_N][ClSeO_2_] from an apparently very accurate 143 K crystal structure [3]. The Se–Cl bond length of 2.453(1) Å in BIRHOZ is significantly longer than that found in **2**, but the apparently equal Se–O distances of 1.632(2) Å agree well with the average of Se1–O1 and Se1–O2 (1.637(1) Å). The accuracy of this report has been confirmed through a personal communication of the structure details (see the Supplementary Materials), so that the divergence between the two geometries will be considered in detail [6]. Krebs et al. further describe this structure in a review article, mentioning that an X-X deformation density analysis has been undertaken on the same salt at 120 K, but these data also remain unpublished: “deformation density maps clearly reveal the presence of lone-pair (E) density (maximum of 0.40 ± 0.04 *e*^−^/Å^−1^ at a distance of ca. 0.75 Å from Se) consistent with model predictions for a pseudo-tetrahedral SO_2_ClE arrangement with additional π density in the Se–O bonds and with a rather polar Se–Cl bond” [4]. From the HAR/NoSpherA2, we were able to extract a deformation density map (Figure 2) that corroborates this verbal description.

### 2.3. Hydrogen Bonding in the Solid Lattice of ***2***

**Figure 3:**
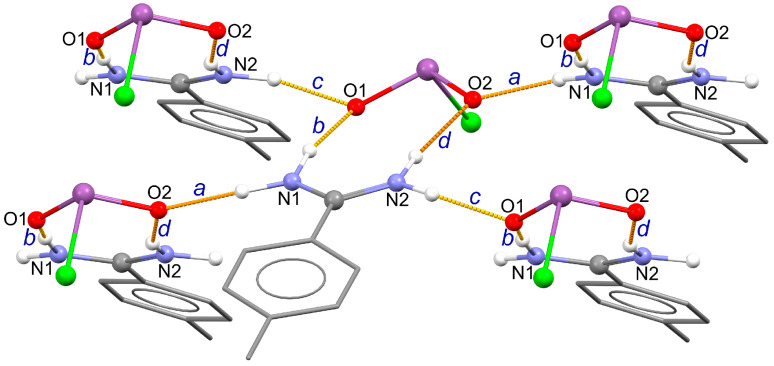
Hydrogen bond network in the crystal lattice of **2**. The Etter notation for the lower-level nets are indicated in the blue lettering: $D_{1}^{1}\left(2\right)$ *a*; $D_{1}^{1}\left(2\right)$ *b*; $D_{1}^{1}\left(2\right)\;$*c*; $D_{1}^{1}\left(2\right)\;$*d*; $C_{2}^{2}\left(6\right)$ > *a* < *b*; $C_{2}^{2}\left(8\right)$ > *a* < *c*; $C_{2}^{1}\left(6\right)$ > *a* < *d*; $C_{2}^{1}\left(6\right)$ > *b* < *c*; $R_{2}^{2}\left(8\right)$ > *b* < *d*; $C_{2}^{2}\left(6\right)$ > *c* < *d*. The relative H-bond strengths are color-coded, with yellow being stronger than orange.

There is an extensive H-bond network (Figure 3, Table 2) in **2** and all the H-bond parameters fit for standard electrostatic-covalent H-bonding according to the criteria of Jeffrey (summarized in Table A1 in Appendix B). The H-bonds form layered nets, wherein every second ion pair is reversed in a head-to-tail fashion that, as viewed in Figure 3, form a ‘Vee’ or roof shape, the horizontal of which aligns with the bifurcator of ∠*ac*. For extended views of these nets, see Figure S3 in the Supplementary Materials. There are both short H-bonds between the amidinium and chloroselenite ions [*d*(D∙∙∙A) 2.852(3) and 2.948(3) Å] and even shorter links to the next amidinium ions on both sides in the net [*d*(D∙∙∙A) 2.852(3) and 2.880(4) Å]. The H-bond acceptor sites at O correspond to negative charge maxima on the computed electrostatic potential surface (Figure S4), which is otherwise unexceptional. Bonds of this length can be worth as much as 50 kJ/mol each (Table A1) so could add up to as much as 200 kJ/mol per formula unit. This is significant stabilization. The primary H-bonds (in Etter notation) are the discrete $D_{1}^{1}\left(2\right)$ links *b* and *d* of the cation to the facing anion, which thereby form an $R_{2}^{2}\left(8\right)$ > *b* < *d* ring, a standard motif for amidinium ions [53]. There are also $D_{1}^{1}\left(2\right)$ links *a* and *c* to two adjacent anions that are of comparable strength to the ring bonds. Many other infinite chain paths and much larger rings can also be identified in the network. Figure 3 also evidences classic π-π stacking, wherein ring carbon atom C3 is closely aligned with the centroid of the phenyl ring below, at a distance of 3.56 Å, with repeats of this interaction throughout the lattice.

### 2.4. Intra-Ionic Short Contacts in the Lattice of ***2***

**Figure 4:**
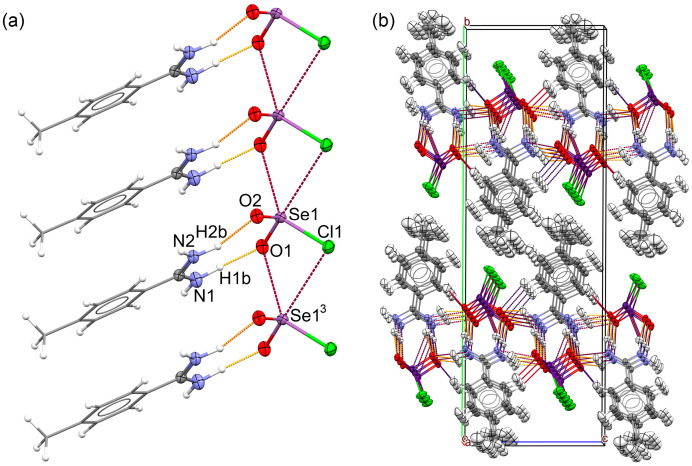
(**a**) The major inter-anion interactions in the crystal lattice of **2**. The chain of ClSeO_2_^−^ ions is aligned with the crystallographic *a* axis. Symmetry code: ^3^ 1 + x,y,z. See Table 3 for metric data. (**b**) Stacked packing diagram viewed down the *a*-axis direction.

We now consider how well the ClSeO_2_^−^ ions in **2** are isolated from each other. Metric parameters for contacts shorter than the sums of the Van der Waals’ radii are included in Table 2. Figure 4a emphasizes the major interactions between ClSeO_2_^−^ ions by including only the ring-forming ion pairs for clarity, whereas Figure 4b shows the overall packing and deliberately includes all atoms to show the intermolecular environment. As is clear from the literature on general oxochlorochalcogenates(IV) [3], there are no truly isolated halochalcogenite ions in crystal lattices, although large organic cations such as PPh_4_^+^ or AsPh_4_^+^ sometimes come close, as in [AsPh_4_][OSeCl_3_] (CSD refcode: BIRGUE10 [54]), which at the very least tend to displace inter-anion contact with more benign donor–acceptor interactions with aromatic ring electron density. With smaller organic cations, inter-anion interactions are commonly observed, both in the forms of discrete dimers and infinite chain polymers. The strongest interactions occur with small monometallic cations, such as in alkali and alkaline earth metal salts, such as the type **C** direct anion chain structure in K[FSeO_2_] [55]. However, many halochalcogenite anions of heavier halogens and chalcogens cannot exist with these small, focused charge, cations.

Specific inter-anion contacts are found in the lattice of **2** (Figure 4a). The anions are doubly bridged by Se1–O1∙∙∙Se1′ and Se1–Cl1∙∙∙Se1′ contacts, forming discrete chains parallel to the crystallographic *a* axis in which the anions are stabilized in three directions by the H-bonds and the large chlorine atoms are surrounded by stacks of tolyl ring methyl groups (Figure 4b). Importantly, the direction of approach of Cl1 to Se1^3^ on the next anion is close to linear with the opposing O1^3^, i.e., the direction consistent with chalcogen bonding from a σ-hole at Se [23]. A consideration of the metrical data in Table 3, specifically the *d*−∑r_vdW_ values, indicates that these inter-anion contacts are relatively weak compared with the anion-cation H-bonding contacts. They are thus comparable (7–8.5% < ∑r_vdW_) to the bridging Cl∙∙∙Se contacts in the chain structure of [Me_4_N][ClSeO_2_] (see Supplementary Materials). This is further borne out by the comparison with the chain-forming Se–Cl∙∙∙Se’ contacts in 8-hydroxyquinolinium trichloro-oxyselenate, [C_9_H_8_NO][OSeCl_3_], which displays *d*−∑r_vdW_ = −0.77 Å (CSD refcode HQNLSE [56]). Other known structures of oxychlorselenium(IV) anion salts, which all show degrees of inter-anion contacts, are as follows: [PPh_4_]_2_[O_2_Se_2_Cl_6_] (CSD refcode: BIRHAL10 [57]); [NEt_4_]_2_[O_2_Se_2_Cl_6_] (CSD refcode: BORCAM [54]); [C_10_H_10_N_2_][OSeCl_4_] (CSD refcode: DPRYSE [58]); [NMe_4_]_3_[O_2_Se_2_Cl_7_][Cl_2_] (CSD refcode: EWILOO [25]); [N*^n^*Pr_4_]_2_[O_2_Se_2_Cl_6_] (CSD refcode: JUCDIU [59] (CSD refcode: RAFYOM [60]).

### 2.5. DFT Computational Investigation of Structure

Surprisingly, no prior computational study of ClSeO_2_^−^ ions could be found in the literature, so it was considered essential to undertake a reliable DFT investigation. Based on precedents in the literature, the B3PW91 functional was selected for its proven efficacy in selenium chemistry [61], but we also chose to enhance it with Grimme’s original D3 dispersion correction to improve its capability for also modelling the full H-bonded salt. For a basis set, aug-CC-pVTZ was selected because of its prior accuracy for selenium compounds [62]. The chosen RB3PW91-D3/aug-CC-pVTZ method was first validated by computing the structure of SeO_2_(g), which provides excellent agreement on geometry and molecular vibrations (see Appendix A).

In a first calculation, the ion pair at the crystal coordinates was computed, which confirms the primary directionality of the H-bonded geometry. A large dipole moment of 15.5 Debye orients almost parallel to the toluamidinium molecular plane and bifurcating the SeO_2_ moiety (Figure 1b). Although far from a complete network, this primary H-bonding (i.e., the $R_{2}^{2}\left(8\right)$ net) does seem to be significantly structure directing (Table 2). Next, the full gas-phase geometry optimization of the ion pair was attempted, which did converge, albeit with a rather more curved overall structure (∠C2∙∙∙Se1–Cl1 = 68.0°) than that found in the lattice geometry (∠C2∙∙∙Se1–Cl1 = 117.0°).

Thereafter, the free ClSeO_2_^−^ ion was geometrically optimized, with full frequency calculations for comparison to the vibrational spectra in early literature reports, when IR, and especially Raman, spectroscopy were used as the chief characterization tools [1,33]. The geometry optimizes to effective *C_s_* point symmetry, as expected, resulting in Se–O lengths of 1.630 Å, close to the average 1.637(1) Å of the two experimental values. Most surprisingly, however, the Se–Cl length *increases* to 2.491 Å, more than 7% longer than the experimental value of 2.3202(9) Å. This is far larger than the expected elongation from just using DFT at this level of theory. Moreover, these values are within the typical DFT accuracy (just 1.5% longer) reported for the BIRHOZ structure on [Me_4_N][ClSeO_2_] [3].

Further support that the shorter Se–Cl length is a real effect from the H-bonding of the toluamidinium to the oxygen atoms of the ClSO_2_^−^ ion in **2** is provided by the above-mentioned gas-phase optimization of the cation-anion pair, where the Se–Cl length remains in the range 2.32 to 2.33 Å through all the optimization steps. Presumably, this is a primarily electrostatic effect, whereby withdrawing ED from the SeO_2_ moiety enhances the presumed dative bond of the Cl^−^ nucleophile in its interaction with Se (see next section). We note that in oxytetrachloroselenate(IV) structures, H-bonding is known to induce *longer* Se–Cl bonds, e.g., in the structure [C_4_H_10_NO][OSeCl_4_] (CSD refcode: RAFYOM [60]) where the Se–Cl elongates to 2.776(2) Å from H-bonding to the morpholinium nitrogen (15% longer than the average of the three other equatorial bonds), or [C_10_H_10_N_2_][OSeCl_4_] (CSD refcode: DPRYSE [58]) where the Se–Cl that is H-bonded to the bipyridinium NH elongates to 2.990(4) Å (24% longer than the equatorial average). Thus, H-bonding to the halogens causes longer Se–Cl bonds, whereas to the oxygen it causes shorter Se–Cl bonds. The observed behavior may also be related to variations in valence sharing.

**Figure 5:**
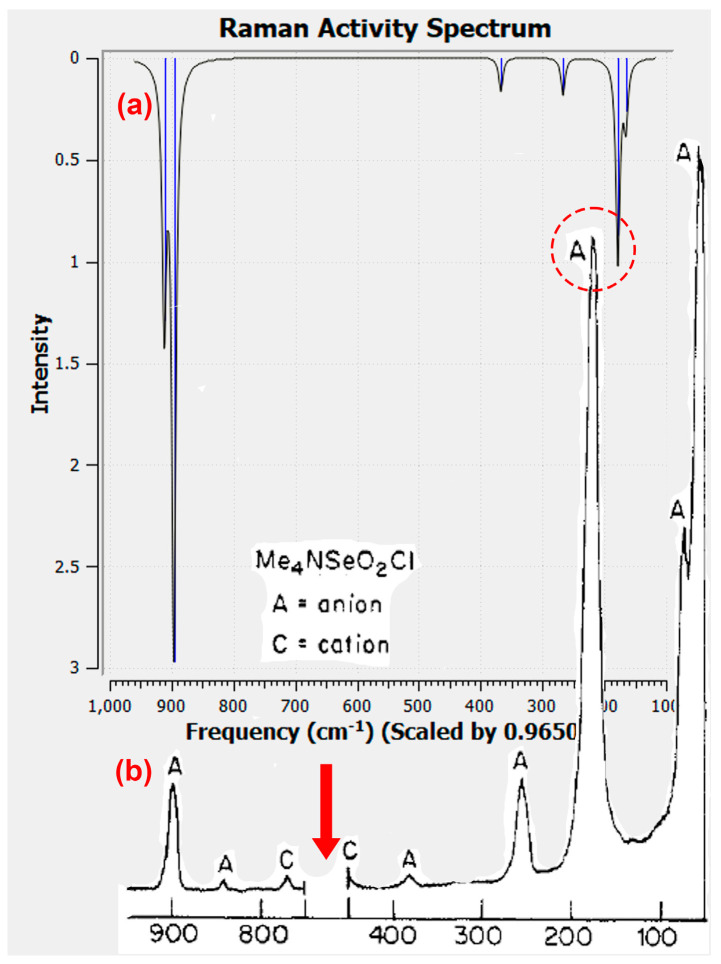
Overlay of (**a**) the RB3PW91-D3/aug-CC-pVTZ DFT Raman spectrum computed on a gas phase isolated ClSeO_2_^−^ anion with (**b**) the experimental spectrum reported on a crystalline powder sample of [Me_4_N][ClSeO_2_]; note that the bottom scale is interrupted at the red arrow, so the band positions must be interpolated. Bands below 100 cm^−1^ are lattice modes. Adapted with permission from LaHaie, P.; Milne, *J. Inorg. Chem.* **1979**, *18*, 632–637 [1]. Copyright (1979) American Chemical Society. Red text is used to distinguish from the original annotations of the underlying graphics.

Seeking further experimental confirmation, we noted that the BIRHOZ structure [3] on [Me_4_N][ClSeO_2_] was one of the very salts originally investigated by Milne using Raman spectroscopy [1]. At that time, the vibrational data were only assigned using symmetry criteria, including experimental depolarization ratios, and by analogy to ClSO_2_^−^ [32]. The results of the RB3PW91-D3/aug-CC-pVTZ-computed spectra, conducted on (i) the optimized gas phase geometry and (ii) on the isolated anion at the X-ray geometry found in **2**, with assignments, and a comparison to the experimental Raman spectroscopy data are compiled in Table 3, and a comparison of one computed and experimental spectrum is shown in Figure 5.

**Figure 6:**
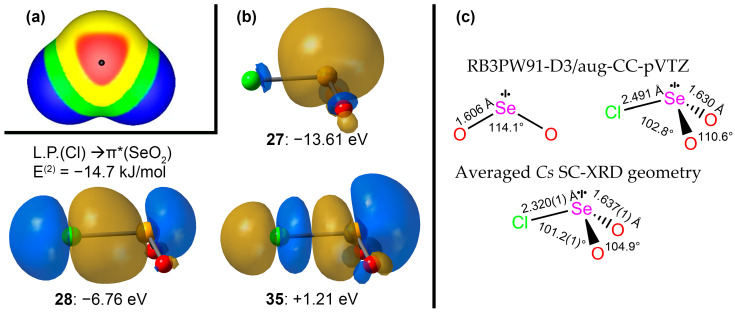
(**a**) Computed electrostatic potential surface at the 0.001 AU level of SeO_2_; the position of the most positive electrostatic potential associated with the π-hole above (and below) Se is indicated by the black hemisphere with V_S,max_ = 148 kJ/mol; adapted with permission from Murray, J. S. et al., *J. Mol. Model.* **2012**, *18*, 541–548 [24]. Copyright (2011) Springer Nature. (**b**) NBO isosurfaces from RB3PW91-D3/aug-CC-pVTZ DFT calculations on gas-phase optimized structure of ClSeO_2_^−^ for (**27**) the Se L.P, (**28**) the Cl L.P. interacting with the empty π*(SeO_2_) orbital; (**35**) the corresponding out-of-phase interaction. Surfaces constructed at the 0.04 AU level. (**c**) Computed geometries (see text).

First, the fit of the numerical data between the experimental and the computed anion values is remarkably good given the combination of experimental uncertainty and comparing gas-phase structures with solids and solutions (there is also very good agreement between the latter two). Importantly, the normal coordinate analysis in the DFT calculations contradict the assignment of ν_2_ to the 200/193 cm^−1^ experimental bands, leading to a switch in the ν_2_ and ν_4_ assignments. A similar discrepancy has already been noted for the vibrational spectra of FSO_2_^−^, which is far and away the most thoroughly studied halochalcogenite anion to date [29]. These workers reported that a normal coordinate analysis with potential energy distribution indicates that the symmetry coordinate of the S–F stretch in FSO_2_^−^ contributes only 20% to ν_2_, and instead has 40% ν_3_ and 39% ν_4_ character. For ClSeO_2_^−^, we find that ν_3_ is not much involved, but there is this formal reversal of ν_2_ and ν_4_, as well as strong coupling that distributes a large amount of S–Cl stretch character to both modes. Viewed through this lens, it is apparent that these two bands are, respectively, 54 and 64 cm^−1^ higher in frequency when computed at the X-ray geometry of **2** than at the DFT-optimized geometry (Table 3 and Figure 6). This, and the good overall fit of the data, is a strong corroboration that the Se–Cl length in [Me_4_N][ClSeO_2_] corresponds to that reported for the BIRHOZ structure [3], and very probably is close to that adopted in CH_3_CN solutions.

### 2.6. Donor–Acceptor Bonding in Hypervalent ClSeO_2_^−^ using the NBO Formalism

After almost a century of debate, a combination of experimental and computational evidence has settled that the bonding in molecular SO_2_ is not hypervalent and corresponds very closely to the description provided by the classical Lewis octet structure with nominal S–O bond orders of 1.5 [63]. SeO_2(g)_ must certainly be described similarly, although under ambient conditions, it forms a solid polymerized via OSeO→Se dative bonding [49]. Indeed, our RB3PW91-D3/aug-CC-pVTZ DFT-computed structure for it has Wiberg bond indices of 1.45 for the Se–O bonds, and 0.31 between the two O atoms. The electrostatic π-holes detected in SeO_2_ (Figure 6a) are oriented above and below the central Se atom and are perpendicular to the molecule plane [24]. Classical nucleophiles such as HCN or NH_3_ are indeed computed to bind to the Se atom close to this perpendicular with interaction distances of 70–89% of the ∑r_vdW_ [24].

Whether or not these π-holes are operative in ClSeO_2_^−^, it is clear from both the experimental and computed geometries that a chloride ion donates electron density to form a kind of dative or charge-transfer bonding, which is stronger than a mere intermolecular interaction, and that is definitely hypervalent [64]. We have applied a natural bond order (NBO) analysis using the NBO 3.1 component within Gaussian W16 on the geometry optimized in the gas phase for ClSeO_2_^−^ (Figure 6b,c). This clearly shows the Se L.P. orbital (#27) very close to co-planar with the SeO_2_ atoms (and hence very similar to that of the educt—see Appendix A). The bond-forming NBO is the Cl L.P.→π*(SeO_2_) orbital (#28), whilst the lowest-energy Rydberg NBO is its out-of-phase companion (#35). The Wiberg bond indices for the optimized geometry are reduced to 1.26 for the Se–O bonds and 0.19 between the O atoms, whilst the bond that forms between Cl and Se has an index of 0.44. This provides an excellent model for weak hyper-valent bonding in the chloroselenite ion and is reminiscent of the bonding models developed for the very well-known trihalide anions [65]. Since Cl→Se bonding has the net effect of occupying the SeO_2_ π* molecular orbital, the π-bond order is expected to be reduced, which rationalizes the lower Wiberg indices for these bonds. And the low bond order for the X–ChO_2_ bond is consistent with the very long X–Ch bond distances in most crystal structures of halochalcogenites and the observation of a wide range of bonding modes, ranging from well-defined XChO_2_^−^ molecular ions to ‘solvated halide’ geometries with almost equal X∙∙∙Ch(O_2_)∙∙∙X determined in some SC-XRD structures (see the Introduction).

Returning now to the chloroselenite structure obtained in our salt **2** with the amidinium ion, it becomes understandable how H-bonding to the two anion oxygen atoms in **2** can have such a strong influence on the Se–Cl bond length, causing it to be more than 5% shorter than observed in the structure of [Me_4_N][ClSeO_2_] (BIRHOZ [3]). In the H-bonded adduct, computed in the gas phase, the NPA charge on Se increases from +1.66 in the optimized ion structure to +1.71, while the charge on Cl decreases from −0.62 to only −0.46. There is thus a clear rationale in the NBO analysis for the shorter Se–Cl bond observed in the structure of **2**, supporting the notion that H-bonding significantly stabilizes the chloroselenite in this amidinium salt.

## 3. Experimental

General synthetic methods for thiazyl and selenazyl chemistry are as previously described [46,66]. The isolation of **4** has been described previously [46].

### 3.1. Chemical Synthesis

Initially, 0.26 g of selenium(IV)-4-NH-dichloroselenatriazine, C_10_H_8_Cl_5_N_3_Se, was exposed to 10 mL of CH_2_Cl_2_ and 10 mL of CH_3_CN and heated in an attempted purification by recrystallization. Upon removing all volatiles, 30 mL of CH_3_CN was added and the mixture was heated to reflux, ensuring all solids were dissolved, and then filtered hot under nitrogen. After cooling, the filtrate was placed in a −10 °C freezer overnight, producing small amounts of solids which were removed by a second filtration. Subsequent cooling of the filtrate in a −30 °C freezer overnight produced well-formed colorless crystals, which were quickly isolated, dried under high vacuum, and submitted for crystallographic study.

### 3.2. Single-Crystal X-ray Crystallography

Suitable crystals were selected under a microscope, mounted on fine glass capillaries in Paratone™ oil, and cooled using the diffractometer cooling wand. Crystal and refinement data are summarized in Table 2. Data for **2** were collected on a Bruker Platform/SMART 1000 CCD diffractometer at the University of Alberta. Image collection, peak identification, cell and space group determination were controlled using SAINT. Multi-scan absorption correction was undertaken using SADABS. Initial structure solution was performed with SHELXS [67]. In view of the extensive H-bonding observed in this structure, refinement was completed in the independent atom model (IAM) using olex2.refine [68] within the Olex2 release 1.5 suite of programs [51]. After a detailed analysis and structure verification in the IAM, Hirsfeld atom refinement was continued using aspherical scattering factors with the aid of NoSpherA2 [50]. A detailed description of our workflow for HAR with aspherical form factors has been published [69].

In the case of **2**, HAR/NoSpherA2 quickly proved to be very successful. The electron density (ED) of each atom was computed using ORCA 5.0 [70] at the R2SCAN/def2-TZVP level of theory, whereafter the custom scattering factors for all atoms were computed using NoSpherA2, and refinement was completed with olex2.refine. When the H-atoms were refined anisotropically, it immediately became apparent that the tolyl methyl group is rotationally disordered, an effect that is normally ignored in IAM where the riding atom approach for H on C usually masks such subtleties. A two-part disorder model was adopted, and HAR resumed (which inter alia requires that two full sets of DFT calculations are required, one for each disorder component). This too proved successful. However, although the overall data quality is quite good (*R*_int_ = 3.28%), the data were obtained on an older sealed-tube diffractometer where the resolution was capped at 0.80 Å. This is probably the origin of the two ghost peaks for the Se atom (Fourier ripples), hindering the overall refinement quality. Hence, in the final refinement cycles the aromatic C-H, distances were restrained to 1.085 Å and N-H distances to 1.040 Å, which are the current best values from neutron refinement in this temperature range [71]. Similar 2- and 3-atom distance restraints were applied to the disordered H atoms in the CH_3_ group, and the occupancies were frozen in a 50:50 ratio. This structure is not intended for validating the performance of the HAR/NoSpherA2 method (which we have previously done thoroughly [69,72,73,74]); instead, it aims for an accurate description of the extensive H-bonding network, and obtaining a deformation density map to show the non-bonded ED.

Crystal Data for C_8_H_11_ClN_2_O_2_Se (*M* = 281.601 g/mol): monoclinic, space group *P*2_1_/*c* (no. 14), *a* = 3.9773(3) Å, *b* = 28.477(2) Å, *c* = 9.5781(9) Å, *β* = 91.242(1)°, *V* = 1084.56(16) Å^3^, *Z* = 4, *T* = 193.15 K, μ(Mo Kα) = 3.685 mm^−1^, *D_calc_* = 1.725 g/cm^3^, 8245 reflections measured (4.48° ≤ 2θ ≤ 52.84°), 2194 unique (*R*_int_ = 0.0328, R_sigma_ = 0.0282) which were used in all calculations. The final *R*_1_ was 0.0339 (I ≥ 2σ(I)) and *wR*_2_ was 0.0827 (all data). CCDC 2301348.

### 3.3. Computational Methods

Initial geometries for the DFT calculations were obtained from the X-ray coordinates (ignoring the second component of the toluene methyl group disorder). All calculations were performed with Gaussian W16 under GUI control from GaussView 6.0 and were run on an AMD Ryzen Threadripper 16-core 3.75 GHz PC under Windows 10 [75]. Minimum energy geometries were verified using harmonic vibrational analysis. The use of Grimme’s D3 correction for dispersion, an attractive effect that is not readily accounted for by the bare B3PW31 functional, was applied in all cases. The suitability of the RB3PW91-D3/aug-CC-pVTZ level of theory was thoroughly investigated by computing the known structural and vibrational properties of SeO_2_ (gas-phase structure), for which, see Appendix A. Atomic charges were computed using normal bond order analysis, and bond orders using the Wiberg definition, all with internal functions. Results computed with Gaussian W16 were visualized and, where required, plotted using GaussView.

## 4. Conclusions

The evidence from this paper is that significant Cl–Se bond shortening is induced in ClSeO_2_^−^ ions by the characteristic H-bonding from benzamidinium cations. This discovery could have major benefits for isolating stable salts of other halochalcogenite(IV) salts. For example, selectivity of the H-bonding for O is considered likely, which may help prevent O/F positional disorder in FChO_2_^−^ salts. These cations may also lead to success in obtaining crystalline adducts for the many X/Ch combinations for which there are no SC-XRD data. This work also emphasizes a point, made previously by others, that work in this area should use multiple, mutually integrated, techniques. For example, vibrational spectroscopy, when fully interpreted by adequate levels of computation, should be feasible on the proposed benzamidinium salts, enhancing the reliability of salt characterization and enabling direct comparisons, especially with Raman spectroscopy, to the structures adopted by these ions in solutions. Additionally, ^77^Se NMR may become invaluable for characterizing the formation of haloselenite ions in solution.

## Figures and Tables

**Table 1:** Interatomic distances (Å) and angles (°) in the crystal structure of **2** and for ClSO_2_^−^ by DFT ^1^.

Atoms	*d*_Experiment_	*d*_Computed_	Atoms	∠_Experiment_	∠_Computed_
Se1–Cl1	2.3202(9)	2.491	O1–Se1–Cl1	100.86(9)	102.75
Se1–O1	1.645(2)	1.630	O2–Se1–Cl1	101.55(9)	102.75
Se1–O2	1.629(2)	1.630	O2–Se1–O1	104.90(11)	110.58
N1–C1	1.323(4)		N2–C1–N1	119.1(3)	
N2–C1	1.317(4)		C2–C1–N1	120.8(3)	
C1–C2	1.472(4)		C2–C1–N2	120.1(3)	
C2–C3	1.404(4)		C3–C2–C1	120.5(3)	
C2–C7	1.395(4)		C7–C2–C3	118.1(3)	
C3–C4	1.382(5)		C4–C3–C2	120.4(3)	
C4–C5	1.395(5)		C5–C4–C3	121.3(3)	
C5–C6	1.392(5)		C6–C5–C4	118.1(3)	
C5–C8	1.497(5)		C8–C5–C4	120.7(3)	
C6–C7	1.384(5)		C8–C5–C6	121.2(3)	
			C7–C2–C1	121.4(3)	
			C7–C6–C5	120.9(3)	

^1^ Full atomic positional and derived data for SC-XRD experiments in the Supplementary Materials. DFT geometry optimized in the gas phase at the RB3PW91-D3/aug-CC-pVTZ level of theory.

**Table 2:** Hydrogen bonds and inter-anion contacts in the crystal structure of **2**.

D(–H)	:A	*d*(D–H)/Å	*d*(H∙∙∙A)/Å	*d*−∑r_vdW_	*d*(D∙∙∙A)/Å	Angle/°	
Hydrogen-bonds							
N1–H1a	O2 ^1^	1.045(17)	1.90(3)	−0.82	2.852(3)	150(3)	
N1–H1b	O1	1.051(18)	1.843(19)	−0.877	2.880(4)	168(3)	
N2–H2a	O1 ^2^	1.030(17)	1.83(3)	−0.89	2.792(3)	153(4)	
N2–H2b	O2	1.039(18)	1.95(2)	−0.77	2.948(3)	160(3)	
Inter-anion Contacts							
O1	Se1 ^3^	(angle is Se1–O1∙∙∙Se1 ^3^)		−0.289	3.131(2)	108.8(1)	
Cl1	Se1 ^3^	(angle is Se1 ^3^–Cl1 ^3^∙∙∙Se1)		−0.252	3.3980(9)	85.96(3)	

Symmetry codes: ^1^ 1 + x,1.5-y,1/2 + z; ^2^ x,1.5-y,-1/2 + z; ^3^ 1 + x,y,z.

**Table 3:** Experimental (Raman) and computed vibrational spectra for ClSO_2_^−^ ions (cm^−1^).

Band	Assignment	Symmetry	Experiment ^1^ CH_3_CN Solution	Experiment ^1^ [Me_4_N][ClSeO_2_]	DFT Optimized ^2^	DFT X-ray Geom. ^3^
ν_1_	ν*_sym_*(SO_2_)	A′	890 (p)	903 (s)	896 (vs,p)	886 (vs,p)
ν_2_	ν(S–Cl)	A′	273 (p) ^4^	267 (s) ^4^	267 (w,p)	321 (m,p)
ν_3_	δ*_sciss_*(SO_2_)	A′	380 (p)	396 (w)	367 (w,p)	382 (w,p)
ν_4_	δ*_sym_*(ClSO_2_^−^)	A′	200 (p) ^4^	193 (vs) ^4^	178 (m,p)	242 (m,p)
ν_5_	ν*_asym_*(SO_2_)	A″	840 (dp)	841 (w)	912 (m,dp)	887 (s,dp)
ν_6_	δ*_asym_*(FSO_2_^−^)	A″	*not obsv.*	*not obsv.*	165 (w,dp)	189 (w,dp)

^1^ As reported in Ref. [1]. ^2^ Frequency calculation of the Raman spectrum with full RB3PW91-D3/aug-CC-pVTZ geometry optimization of the anion geometry. ^3^ RB3PW91-D3/aug-CC-pVTZ-computed anion at the X-ray geometry in the crystal lattice of **2**, symmetrized to *Cs*. ^4^ Reversals of the 1978 assignments, based on the vibrational symmetries obtained by DFT; the colored bands draw attention to this switch. Notably, the ν_2_ and ν_4_ bands are strongly coupled, and hence both will reflect variations in S–Cl bond strength.

**Figure A1:**
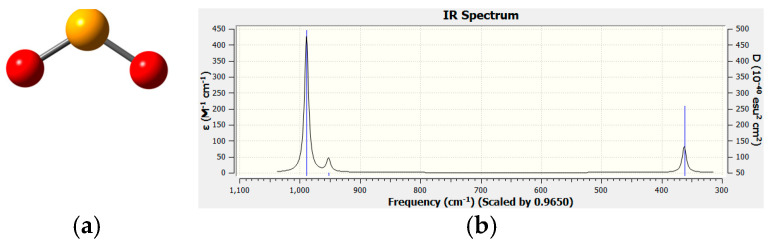
(**a**) Structure of SeO_2_ (in the gas phase; it is a polymeric solid at RT). (**b**) Computed IR spectrum at the RB3PW91-D3/aug-CC-pVTZ level of theory.

**Figure A2:**
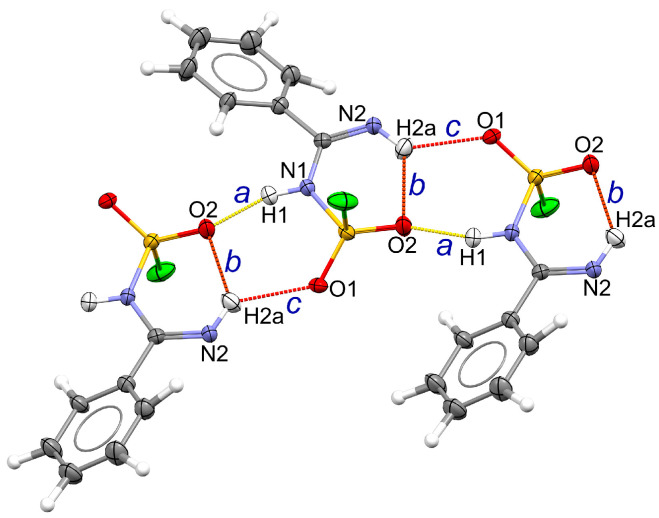
Hydrogen bond network in the crystal lattice of **4** (structure taken from CSD refcode EZOWUM). The Etter notation for the lower-level nets are indicated in the blue lettering: $C_{1}^{1}\left(4\right)$ *a*; $S_{1}^{1}\left(6\right)$ *b*; $C_{1}^{1}\left(6\right)\;$*c*; $C_{2}^{2}\left(8\right)$ > *a* < *c*; $R_{2}^{2}\left(10\right)$ > *a* > *c*. The relative H-bond strengths are color-coded with yellow stronger than red.

## Data Availability

CCDC 2301348 contains the supplementary crystallographic data for this paper. These data can be obtained free of charge via www.ccdc.cam.ac.uk/data_request/cif (accessed on 14 October 2023), or by emailing data_request@ccdc.cam.ac.uk, or by contacting The Cambridge Crystallographic Data Centre, 12 Union Road, Cambridge CB2 1EZ, UK; fax: +44-1223-336033 or from the Inorganic Crystal Structure Database, https://icsd.products.fiz-karlsruhe.de/ accessed on 14 October 2023.

## Supplementary Materials

The following supporting information can be downloaded at: https://www.mdpi.com/article/10.3390/molecules28227489/s1, Supplementary report, with structural details on BIRHOZ, further information on **2** and a full crystal structure report for **2**. References [6,50,51,67,68] are cited in the Supplementary Materials.

## Appendix A

Computational method calibration: SeO_2_ at the RB3PW91-D3/aug-CC-pVTZ level of theory computes *d*(Se–O) = 1.6026 Å; ∠(OSeO) 114.1°(cf. 1.6076(6); 113.83(8)° from microwave spectroscopy [76]). Vibrational spectrum: ν_2_″ 362 cm^−1^; ν_3_″ 989 cm^−1^ (cf. 364; 968 cm^−1^ in the gas phase above solid SeO_2_, >350 °C by infra-red spectroscopy [77]). This gives high confidence in the method so that agreement with experimental data for ClSeO_2_^−^ will reflect accurately the structural data for this weakly bound ion.

The MO sequence (1*a*_1_)^2^(1*b*_2_)^2^(2*a*_1_)^2^(2*b*_2_)^2^(3*a*_1_)^2^(1*b*_1_)^2^(1*a*_2_)^2^(3*b*_2_)^2^(4*a*_1_)^2^(2*b*_1_)^0^ computed at our level is in remarkable agreement with Walsh’s original theoretical predictions for AB_2_ molecules at a bond angle of 114° [78], being (1*a*_1_)^2^(1*b*_2_)^2^(2*a*_1_)^2^(1*b*_1_)^2^(3*a*_1_)^2^(2*b*_2_) ^2^(1*a*_2_)^2^(3*b*_2_)^2^(4*a*_1_)^2^(2*b*_1_)^0^, so that only 1*b*_1_ and 2*b*_2_ levels are exchanged (the π_1_ MO is lower-lying in the generic Walsh interaction diagram, probably reflecting stronger π-overlap in the model system compared to that of the 4th period Se). Moreover, there is good agreement in the relative energy levels for the two highest filled and lowest virtual levels, such that the HOMO is the in-plane sulfur L.P. orbital by both approaches. Importantly for anion coordination, as in [ClSO_2_]^−^, the lowest unoccupied MO of SeO_2_ is unambiguously the 2*b*_1_ π3* orbital dominated by the empty Se 4*p*_z_ AO.

## Appendix B

The molecular and crystal structure of **4** has been reported previously in the supplementary data of Ref. [46] and is available from the CSD via refcode EZOWUM (or via its acquisition code: CCDC 851053). Allowing for the differing covalent radii of S and Se (1.02; 1.16 Å), the bond lengths at the chalcogen seem quite comparable to those in the ClSeO_2_^−^ ion in **2**, another indication of the relatively strong Se–Cl bond in that structure. However, the angles at S in sulfamyl chloride **4** are closer to tetrahedral values, as expected for a four-coordinate structure. Almost all the bonds and angles around the S atom in **4** fit well with the averages from 20 neutral comparator structures of other sulfamyl chlorides from a CSD search. The exception is *d*(S1–N1) which at 1.695(3) Å is 7% longer than the average of 1.59(3) Å. However, the average when restricted to just those ten comparators in which the attachment atom is not double bonded (to C or especially P) is closer, at 1.629(3) Å.

The H-bonds found in the lattice of **4** (Figure A2) are of only three types, but they are found to be slightly shorter even than those in **2**, and hence are expected to total about 150 kJ/mol in stabilization energy. The Etter notation for the lower-level nets are shown in the figure using blue lettering. Evidently, H-bonding is well able to stabilize this very reactive sulfamyl chloride.

Table A1 is a very useful compilation of H-bond properties, which enables a meaningful interpretation of the data found in the structures of **2** and **4**, as well as many other structures. These data have been compiled by the author from various literature sources and modified to be suitable for acceptors from both the second and the third periods of the main group elements.

**Table A1:** Ranking of Hydrogen Bond Strengths and Properties Adapted from Jeffrey *.

Parameter	Strong	Moderate	Weak
Interaction type:	strongly covalent	mostly electrostatic	electrostatic/dispersion
*d*(H···A), Å ^#^	1.2–1.5	1.5–2.2	>2.2
*d*(D···A), Å ^#^	2.2–2.5	2.5–3.2	>3.2
*lengthening* of D–H	0.08–0.25	0.02–0.08	<0.02
D–H *versus* H···A	D–H ≈ H···A	D–H < H···A	D–H << H···A
Directionality:	Strong	moderate	weak
∠D–H···A, °	170–180	>130	>90
Bond energy, kJ mol^−1^	60–160	16–60	<16
IR shift $\Delta {\bar{\nu }}_{DH}$, cm^−1^	25%	10–25%	<10%
^1^H downfield shift, ppm	14–22	<14	

* Jeffrey, G. A. *An introduction to hydrogen bonding*. Oxford University Press: New York, 1997, Volume 12, pp. 330 [79]. ^#^ For a given donor type, the hydrogen-bond distance typically increases by over 0.5 Å from 2nd to 3rd period, 0.15 Å from 4th to 5th period, and 0.25 Å from 5th to 6th period acceptors [80].
