# Supplementary material for: Hydrogen Bonds Stabilize Chloroselenite Anions: Crystal Structure of a New Salt and Donor-Acceptor Bonding to SeO2
Source: Molecules. 2023 Nov 8;28(22):7489. doi: 10.3390/molecules28227489 (PMC10673179; doi:10.3390/molecules28227489)
Supplement: Supplementary file 1 [file molecules-28-07489-s001.zip › 2_Tolylamidinium_ClSeO2_cifreport.pdf]

checkCIF/PLATON report

Structure factors have been supplied for datablock(s) 2_Tolylamidinium_ClSeO2

THIS REPORT IS FOR GUIDANCE ONLY. IF USED AS PART OF A REVIEW PROCEDURE FOR PUBLICATION, IT SHOULD NOT REPLACE THE EXPERTISE OF AN EXPERIENCED CRYSTALLOGRAPHIC REFEREE.

No syntax errors found. CIF dictionary Interpreting this report

Datablock: 2_Tolylamidinium_ClSeO2

Bond precision: C-C = 0.0046 A Wavelength=0.71073

Cell:	a=3.9773(3)	b=28.477(2)	c=9.5781(9)
	alpha=90	beta=91.242(1)	gamma=90
Temperature:	193 K		

	Calculated	Reported
Volume	1084.58(15)	1084.56(16)
Space group	P 21/c	P 1 21/c 1
Hall group	-P 2ybc	-P 2ybc
Moiety formula	C8 H11 N2, Cl O2 Se	Cl O2 Se, C8 H11 N2
Sum formula	C8 H11 Cl N2 O2 Se	C8 H11 Cl N2 O2 Se
Mr	281.60	281.60
Dx,g cm-3	1.725	1.725
Z	4	4
Mu (mm-1)	3.685	3.685
F000	560.0	560.8
F000'	560.42	
h,k,lmax	4,35,11	4,35,11
Nref	2198	2194
Tmin,Tmax	0.276,0.643	0.350,0.666
Tmin'	0.255	

Correction method= # Reported T Limits: Tmin=0.350 Tmax=0.666
AbsCorr = MULTI-SCAN

Data completeness= 0.998 Theta(max)= 26.420

R(reflections)= 0.0339(1967) wR2(reflections)= 0.0827(2194)

S = 1.103 Npar= 253

```
The following ALERTS were generated. Each ALERT has the format
       test-name_ALERT_alert-type_alert-level.
Click on the hyperlinks for more details of the test.
```

Alert level C

```
PLAT042_ALERT_1_C Calc. and Reported MoietyFormula Strings  Differ     Please Check
PLAT088_ALERT_3_C Poor Data / Parameter Ratio ....................       8.67 Note
PLAT245_ALERT_2_C U(iso) H8E       Smaller than U(eq) C8        by      0.023 Ang**2
PLAT353_ALERT_3_C Long   N-H (N0.87,N1.01A)  N1       - H1A      .       1.04 Ang.
PLAT353_ALERT_3_C Long   N-H (N0.87,N1.01A)  N1       - H1B      .       1.05 Ang.
PLAT353_ALERT_3_C Long   N-H (N0.87,N1.01A)  N2       - H2A      .       1.03 Ang.
PLAT353_ALERT_3_C Long   N-H (N0.87,N1.01A)  N2       - H2B      .       1.04 Ang.
PLAT414_ALERT_2_C Short Intra D-H..H-X       H1A      ..H7       .       1.92 Ang.
                                                      x,y,z  =      1_555 Check
PLAT414_ALERT_2_C Short Intra D-H..H-X       H2A      ..H3       .       1.90 Ang.
                                                      x,y,z  =      1_555 Check
PLAT906_ALERT_3_C Large K Value in the Analysis of Variance ......      2.778 Check
```

Alert level G

```
PLAT002_ALERT_2_G Number of Distance or Angle Restraints on AtSite         21 Note
PLAT003_ALERT_2_G Number of Uiso or Uij Restrained non-H Atoms ...          6 Report
PLAT005_ALERT_5_G No Embedded Refinement Details Found  in the CIF     Please Do !
PLAT068_ALERT_1_G Reported F000 Differs from Calcd (or Missing)...     Please Check
PLAT164_ALERT_4_G Nr. of Refined C-H H-Atoms in Heavy-Atom Struct.          4 Note
PLAT300_ALERT_4_G Atom Site Occupancy of H8A        Constrained at        0.5 Check
PLAT300_ALERT_4_G Atom Site Occupancy of H8B        Constrained at        0.5 Check
PLAT300_ALERT_4_G Atom Site Occupancy of H8C        Constrained at        0.5 Check
PLAT300_ALERT_4_G Atom Site Occupancy of H8D        Constrained at        0.5 Check
PLAT300_ALERT_4_G Atom Site Occupancy of H8E        Constrained at        0.5 Check
PLAT300_ALERT_4_G Atom Site Occupancy of H8F        Constrained at        0.5 Check
PLAT779_ALERT_4_G Suspect or Irrelevant (Bond) Angle(s) in CIF ...      38.00 Deg.
              H8D  -C8   -H8C     1_555   1_555   1_555 ......     #   40 Check
PLAT779_ALERT_4_G Suspect or Irrelevant (Bond) Angle(s) in CIF ...      41.00 Deg.
              H8F  -C8   -H8B     1_555   1_555   1_555 ......     #   48 Check
PLAT790_ALERT_4_G Centre of Gravity not Within Unit Cell: Resd.  #          2 Note
              Cl O2 Se
PLAT802_ALERT_4_G CIF Input Record(s) with more than 80 Characters          1 Info
PLAT860_ALERT_3_G Number of Least-Squares Restraints .............        143 Note
PLAT910_ALERT_3_G Missing # of FCF Reflection(s) Below Theta(Min).          1 Note
PLAT912_ALERT_4_G Missing # of FCF Reflections Above STh/L=  0.600          4 Note
PLAT978_ALERT_2_G Number C-C Bonds with Positive Residual Density.          8 Info
PLAT979_ALERT_1_G NoSpherA2 Scattering Factors Used .............      Please Note
```

```
   0 ALERT level A = Most likely a serious problem - resolve or explain
   0 ALERT level B = A potentially serious problem, consider carefully
  10 ALERT level C = Check. Ensure it is not caused by an omission or oversight
  20 ALERT level G = General information/check it is not something unexpected

   3 ALERT type 1 CIF construction/syntax error, inconsistent or missing data
   6 ALERT type 2 Indicator that the structure model may be wrong or deficient
   8 ALERT type 3 Indicator that the structure quality may be low
  12 ALERT type 4 Improvement, methodology, query or suggestion
   1 ALERT type 5 Informative message, check
```

It is advisable to attempt to resolve as many as possible of the alerts in all categories. Often the minor alerts point to easily fixed oversights, errors and omissions in your CIF or refinement strategy, so attention to these fine details can be worthwhile. In order to resolve some of the more serious problems it may be necessary to carry out additional measurements or structure refinements. However, the purpose of your study may justify the reported deviations and the more serious of these should normally be commented upon in the discussion or experimental section of a paper or in the "special_details" fields of the CIF. checkCIF was carefully designed to identify outliers and unusual parameters, but every test has its limitations and alerts that are not important in a particular case may appear. Conversely, the absence of alerts does not guarantee there are no aspects of the results needing attention. It is up to the individual to critically assess their own results and, if necessary, seek expert advice.

Publication of your CIF in IUCr journals

A basic structural check has been run on your CIF. These basic checks will be run on all CIFs submitted for publication in IUCr journals (*Acta Crystallographica*, *Journal of Applied Crystallography*, *Journal of Synchrotron Radiation*); however, if you intend to submit to *Acta Crystallographica Section C* or *E* or *IUCrData*, you should make sure that full publication checks are run on the final version of your CIF prior to submission.

Publication of your CIF in other journals

Please refer to the *Notes for Authors* of the relevant journal for any special instructions relating to CIF submission.

PLATON version of 06/07/2023; check.def file version of 30/06/2023
