# Supplementary material for: Hydrogen Bonds Stabilize Chloroselenite Anions: Crystal Structure of a New Salt and Donor-Acceptor Bonding to SeO2
Source: Molecules. 2023 Nov 8;28(22):7489. doi: 10.3390/molecules28227489 (PMC10673179; doi:10.3390/molecules28227489)
Supplement: Supplementary file 1 [file molecules-28-07489-s001.zip › Supplementary Report for the Paper R1.pdf]

Hydrogen Bonds Stabilize Chloroselenite Anions: Crystal Structure of a New Salt and Donor-Acceptor Bonding to SeO_2

René T. Boeré

Supplementary Materials

Table of Contents Page

1. Structure Information on $[Me_4N][ClSeO_2]$ S2

2. Further information on **2** and the anion $ClSeO_2^-$ S3

3. Full Crystal Structure Report for the salt **2** S4

4. Additional References for the Supplementary Information S8

1. Structure Information on [Me_4N][$ClSeO_2$]

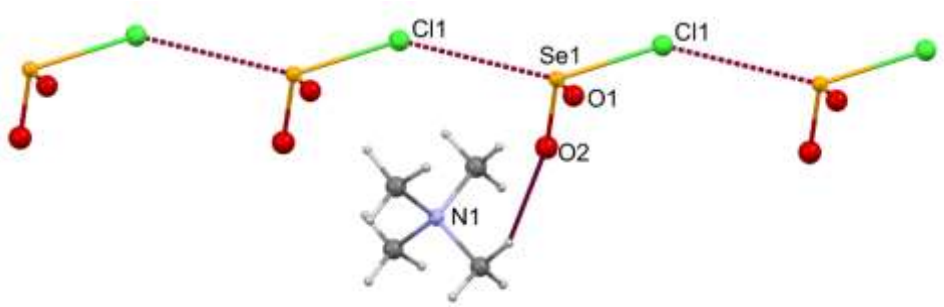

Figure S1. A representation (ball & stick model) of the crystal structure of [Me_4N][$ClSeO_2$] (CSD refcode BIRHOZ). The atom coordinates were communicated personally by Prof. B. Krebs [S1].

The lattice for [Me_4N][$ClSeO_2$] (Figure S1) consists of columns of catenated $ClSeO_2^-$ anions that are weakly bridged, with each one also weakly (non-classical) H-bonded to a methyl group of the cation. The chains align with the unit cell *a* axis (Figure S2). The two Se–O bonds are equivalent in length within statistical certainty but not symmetry-equivalent. The 'bonded' Se–Cl length at 2.453(1) Å is 14% longer than the sums of covalent radii, but 1.197 Å (33%) less than the sums of van der Waals' radii. The 'bridging' Se–Cl length at 3.369(2) Å is 60% longer than the sums of covalent radii, but only 0.281 Å (7.7%) less than the sums of van der Waals' radii. Hence, this is a relatively weak intermolecular contact, quite similar to and even slightly shorter than, the inter-anion contacts found in the crystal structure of **2** in our work. In the structure of K[$FSeO_2$], which displays a quite similar extended chain with short 'bonds' and longer 'contacts', the former are 47% < $\sum r_{vdW}$ while the latter are as much as 16% < $\sum r_{vdW}$ [54]. So it is a bridged structure, making it formally of type **C**, but the bridges are so weak that it remains *effectively* type **A**, as we have described in in the Article. By corollary, the bridging O···Se and Cl···Se contacts in **2** should not be expected to significantly affect the bonding in the anion.

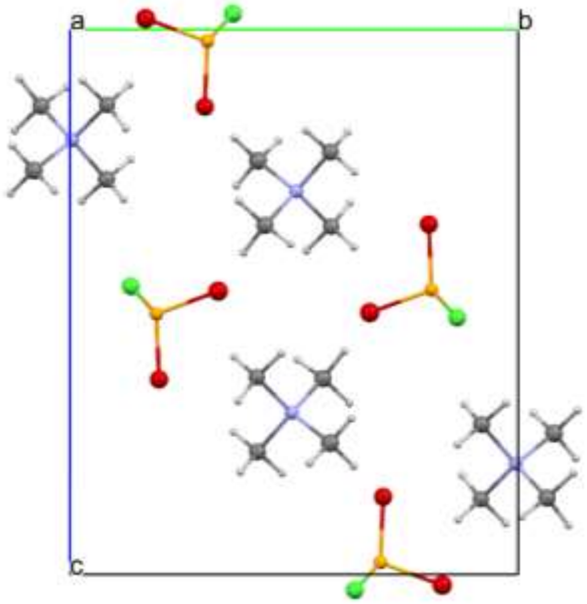

Figure S2. Packing diagram viewed down the *a* direction.

We thank Prof. Dr. B. Krebs for personal communication of the atom coordinates and other structure details that remain unpublished for [Me_4N][$ClSeO_2$], see ref [S1].

2. Further information on 2 and the anion $ClSeO_2^-$

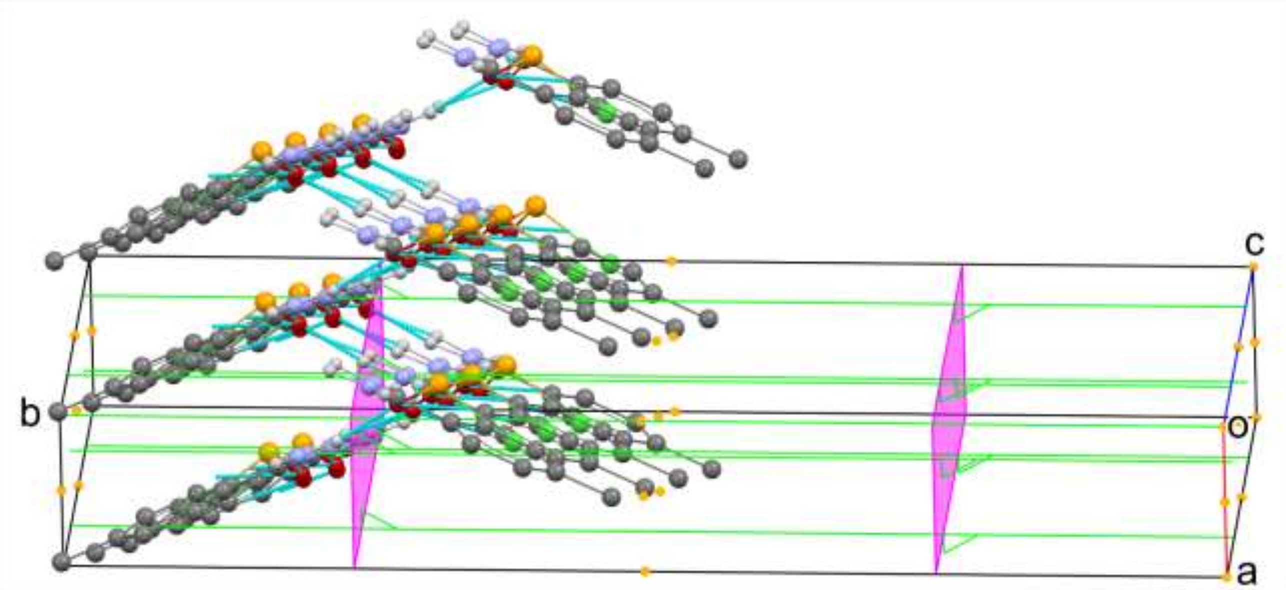

Figure S3. Ball and stick model of the crystal structure of **2** showing the unit cell, lattice symmetry elements and the 'inverted V' or roofline structure of the H-bonded nets. The view is down the bifurcator of bifurcator of $\angle ac$. By inversion symmetry, an opposite 'V' stack is found at the r.h.s. to complete the unit cell filling.

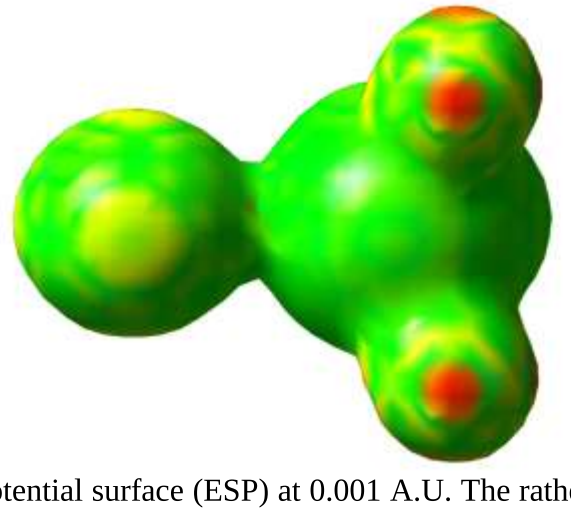

Figure S4. Electrostatic potential surface (ESP) at 0.001 A.U. The rather featureless surface shows both the weak overlap between Cl (at left) and the SeO_2 moiety (at right) and the negative charge accumulation a O consistent with the dual H-bond acceptor role of each O in the H-bonded net (Figures 3 and S3).

3. Full Crystal Structure Report for the salt 2

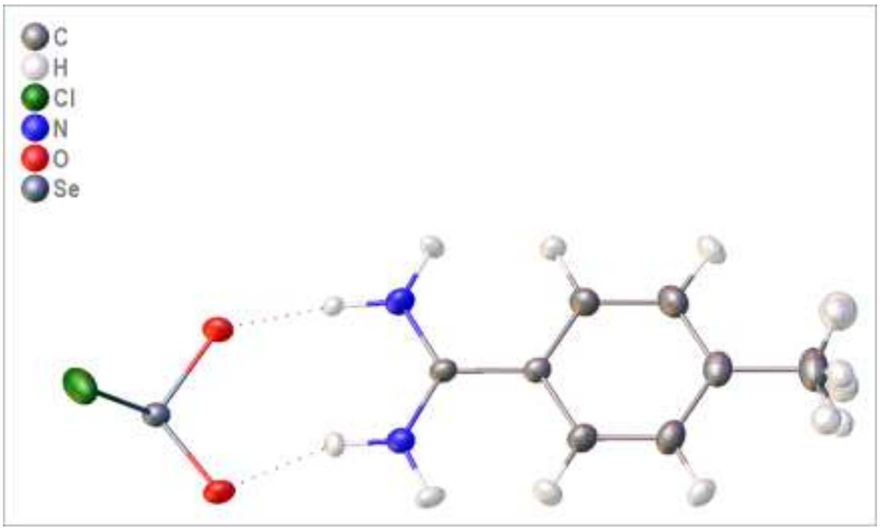

Table S1 Crystal data and structure refinement for 2.

Identification code	let0507b
Empirical formula	$C_8H_{11}ClN_2O_2Se$
Formula weight	281.601
Temperature/K	193.15
Crystal system	monoclinic
Space group	$P2_1/c$
a/Å	3.9773(3)
b/Å	28.477(2)
c/Å	9.5781(9)
α/°	90
β/°	91.242(1)
γ/°	90
Volume/Å^3	1084.56(16)
Z	4
ρ_{calc}g/cm^3	1.725
μ/mm^{-1}	3.685
F(000)	560.8
Crystal size/mm^3	0.36 × 0.3 × 0.12
Radiation	Mo Kα (λ = 0.71073)
2Θ range for data collection/°	4.48 to 52.84
Index ranges	$-4 \leq h \leq 4$, $-35 \leq k \leq 35$, $-11 \leq l \leq 11$
Reflections collected	8245
Independent reflections	2194 [R_{int} = 0.0328, R_{sigma} = 0.0282]
Data/restraints/parameters	2194/149/253
Goodness-of-fit on F^2	1.106
Final R indexes [I>=2σ (I)]	R_1 = 0.0340, wR_2 = 0.0807
Final R indexes [all data]	R_1 = 0.0394, wR_2 = 0.0829
Largest diff. peak/hole / e Å^{-3}	1.06/-0.76

Table S2 Fractional Atomic Coordinates (×10^4) and Equivalent Isotropic Displacement Parameters (Å^2×10^3) for 2. U_{eq} is defined as 1/3 of the trace of the orthogonalised U_{IJ} tensor.

Atom	*x*	*y*	*z*	U(eq)
Se1	-3968.1(8)	6691.0(1)	8440.2(3)	24.03(12)
Cl1	-954(2)	6041.4(3)	7710.5(11)	44.7(2)
O1	-1158(6)	6950.2(8)	9462(2)	32.4(5)
O2	-4109(6)	7008.3(8)	7025(2)	34.4(5)
N1	1940(8)	7864.1(10)	9533(3)	33.0(6)
N2	-263(7)	7889.0(9)	7321(3)	27.8(6)
C1	1489(7)	8091.1(11)	8338(3)	22.4(6)
C2	2921(7)	8562.3(10)	8138(3)	22.4(6)
C3	2929(9)	8767.9(12)	6806(3)	30.2(7)
C4	4267(9)	9211.1(13)	6621(4)	36.9(8)
C5	5698(8)	9461.5(12)	7736(4)	34.8(8)
C6	5693(10)	9256.4(13)	9055(4)	39.8(8)
C7	4334(9)	8814.7(12)	9253(3)	33.7(7)
C8	7120(11)	9942.2(14)	7519(5)	49.6(10)

Table S3 Anisotropic Displacement Parameters (Å^2×10^3) for 2. The Anisotropic displacement factor exponent takes the form: $-2\pi^2[h^2a^{*2}U_{11}+2hka^*b^*U_{12}+\ldots]$.

Atom	U_{11}	U_{22}	U_{33}	U_{12}	U_{13}	U_{23}
Se1	25.49(18)	27.28(18)	19.18(17)	-0.38(12)	-2.45(11)	-0.01(12)
Cl1	39.9(5)	31.5(4)	62.2(6)	4.3(4)	-10.0(4)	-14.6(4)
O1	47.2(15)	30.8(12)	18.6(10)	-3.5(10)	-10.0(9)	-2.7(9)
O2	48.2(15)	34.2(12)	20.3(11)	-3.0(10)	-12.2(10)	4.0(9)
N1	50.0(18)	30.8(15)	18.1(13)	-6.0(13)	-6.3(12)	3.6(11)
H1a	42(15)	48(16)	17(9)	-14(7)	2(5)	-1(5)
H1b	38(15)	28(7)	21(16)	0(4)	-3(8)	2(4)
N2	37.3(16)	25.3(14)	20.6(13)	-0.8(12)	-6.0(11)	-0.8(10)
H2a	56(19)	64(18)	34(7)	-1(8)	-10(5)	19(5)
H2b	45(17)	27(6)	35(17)	-3(4)	0(8)	-2(4)
C1	28.7(16)	24.1(15)	14.4(13)	1.3(12)	-1.0(11)	0.0(11)
C2	26.5(15)	25.3(15)	15.4(14)	0.7(12)	2.0(11)	0.3(11)
C3	39.3(18)	30.7(17)	20.6(15)	-1.9(14)	1.1(12)	2.2(12)
H3	60(16)	58(16)	33(10)	-12(7)	-3(5)	-10(5)
C4	47(2)	30.0(17)	33.6(18)	-3.3(15)	2.9(15)	6.8(14)
H4	69(18)	55(17)	40(6)	-2(8)	3(4)	17(4)
C5	31.5(18)	28.3(17)	45(2)	0.5(14)	4.5(14)	2.1(15)
C6	49(2)	31.2(18)	39(2)	-5.6(15)	-9.3(16)	-4.7(15)
H6	55(17)	58(15)	46(11)	-15(7)	-5(6)	-16(6)
C7	47(2)	29.3(17)	24.8(16)	-4.4(15)	-7.4(14)	-2.4(13)
H7	68(18)	45(16)	29(6)	-10(8)	-9(4)	3(4)
C8	44(2)	31(2)	74(3)	-4.2(17)	2(2)	3(2)
H8a	90(30)	90(30)	100(30)	-1(9)	2(9)	0(9)
H8b	50(30)	50(30)	60(30)	0(9)	-1(9)	-4(9)

Table S3 Anisotropic Displacement Parameters (Å^2×10^3) for 2. The Anisotropic displacement factor exponent takes the form: $-2\pi^2[h^2a^{*2}U_{11}+2hka^*b^*U_{12}+…]$.

Atom	U_{11}	U_{22}	U_{33}	U_{12}	U_{13}	U_{23}
H8c	50(30)	40(20)	50(30)	0(9)	-1(9)	-7(9)
H8d	60(30)	60(30)	60(30)	0(9)	3(9)	-2(9)
H8e	30(20)	50(20)	40(20)	-1(9)	2(9)	3(9)
H8f	60(30)	60(30)	60(30)	1(9)	1(9)	3(9)

Table S4 Bond Distances for 2.

Atoms	Length/Å	Atoms	Length/Å
Se1–Cl1	2.3202(9)	O1–Se1–Cl1	100.86(9)
Se1–O1	1.645(2)	O2–Se1–Cl1	101.55(9)
Se1–O2	1.629(2)	O2–Se1–O1	104.90(11)
N1–C1	1.323(4)	N2 C1 N1	119.1(3)
N2–C1	1.317(4)	C2 C1 N1	120.8(3)
C1–C2	1.472(4)	C2 C1 N2	120.1(3)
C2–C3	1.404(4)	C7 C2 C3	118.1(3)
C2–C7	1.395(4)	C4 C3 C2	120.4(3)
C3–C4	1.382(5)	C5 C4 C3	121.3(3)
C4–C5	1.395(5)	C6 C5 C4	118.1(3)
C5–C6	1.392(5)	C8 C5 C4	120.7(3)
C5–C8	1.497(5)	C8 C5 C6	121.2(3)
C6–C7	1.384(5)	C7 C2 C1	121.4(3)
C3–C2–C1	120.5(3)	C7 C6 C5	120.9(3)

Table S5 Bond Angles for 2.

Atom	Atom	Atom	Angle/°	Atom	Atom	Atom	Angle/°
O1	Se1	Cl1	100.86(9)	C7	C2	C3	118.2(3)
O2	Se1	Cl1	101.56(9)	C4	C3	C2	120.4(3)
O2	Se1	O1	104.90(11)	C5	C4	C3	121.4(3)
N2	C1	N1	119.1(3)	C6	C5	C4	118.2(3)
C2	C1	N1	120.8(3)	C8	C5	C4	120.7(3)
C2	C1	N2	120.1(3)	C8	C5	C6	121.1(4)
C3	C2	C1	120.5(3)	C7	C6	C5	120.9(3)
C7	C2	C1	121.4(3)	C6	C7	C2	121.0(3)

Table S6 Hydrogen Bonds for 2.

D	H	A	d(D-H)/Å	d(H-A)/Å	d(D-A)/Å	D-H-A/°
N1	H1a	O2[1]	1.045(17)	1.90(3)	2.852(3)	150(3)
N1	H1b	O1	1.051(18)	1.843(19)	2.880(4)	168(3)
N2	H2a	O1[2]	1.030(17)	1.83(3)	2.792(3)	153(4)

[1]1+X,3/2-Y,1/2+Z; [2]+X,3/2-Y,-1/2+Z

Table S7 Torsion Angles for 2.

A	B	C	D	Angle/°	A	B	C	D	Angle/°
N1	C1	C2	C3	169.2(3)	C2	C3	C4	C5	1.3(4)
N1	C1	C2	C7	-10.1(4)	C2	C7	C6	C5	-0.2(4)
N2	C1	C2	C3	-10.7(4)	C3	C4	C5	C6	-1.1(4)
N2	C1	C2	C7	170.0(3)	C3	C4	C5	C8	-179.7(4)
C1	C2	C3	C4	179.8(3)	C4	C5	C6	C7	0.6(4)
C1	C2	C7	C6	179.6(3)					

Table S8 Hydrogen Atom Coordinates (Å×10⁴) and Isotropic Displacement Parameters (Å²×10³) for 2.

Atom	*x*	*y*	*z*	U(eq)
H1a	3150(90)	8027(13)	10380(30)	36(9)
H1b	750(90)	7537(9)	9650(40)	29(9)
H2a	-780(110)	8048(16)	6370(30)	52(11)
H2b	-1240(100)	7551(8)	7380(40)	36(9)
H3	1810(100)	8584(13)	5920(30)	50(10)
H4	4130(110)	9354(14)	5580(20)	54(10)
H6	6820(100)	9462(13)	9890(30)	53(10)
H7	4350(100)	8676(13)	10320(20)	48(9)
H8a	8100(200)	10080(30)	8500(50)	90(30)
H8b	9130(150)	9960(30)	6770(70)	50(20)
H8c	5240(140)	10190(20)	7140(80)	50(20)
H8d	5500(170)	10220(20)	7850(90)	60(20)
H8e	9660(80)	9960(30)	7940(70)	39(18)
H8f	7400(200)	9950(30)	6392(16)	60(20)

Table S9 Atomic Occupancy for 2.

Atom	*Occupancy*	Atom	*Occupancy*	Atom	*Occupancy*
H8a	0.500000	H8b	0.500000	H8c	0.500000
H8d	0.500000	H8e	0.500000	H8f	0.500000

Experimental

Single crystals of $C_8H_{11}ClN_2O_2Se$ **2** were grown from acetonitrile at -30°C. A suitable crystal was selected and mounted on a fine glass capillary with Paratone oil and placed on a Bruker PLATFORM on a Smart 1000 CCD area detector diffractometer. The crystal was kept at 193.15 K during data collection. Using Olex2 [S2], the structure was solved with the SHELXS [S3] structure solution program using Direct Methods and refined with the olex2.refine [S4] refinement package using Gauss-Newton minimization. Refinement using NoSpherA2, an implementation of NOn-SPHERical Atom-form-factors in Olex2 [S5]. NoSpherA2 implementation of HAR makes use of tailor-made aspherical atomic form factors calculated on-the-fly from a Hirshfeld-partitioned electron density (ED) - not from spherical-atom form factors.

The ED is calculated from a gaussian basis set single determinant SCF wavefunction - either Hartree-Fock or DFT using selected functionals - for a fragment of the crystal. This fragment can be embedded in an electrostatic crystal field by employing cluster charges or modelled using implicit solvation models, depending on the software used.

The following options were used:

SOFTWARE: ORCA 5.0
PARTITIONING: NoSpherA2
INT ACCURACY: Normal
METHOD: R2SCAN
BASIS SET: def2-TZVP
CHARGE: 0
MULTIPLICITY: 1
DATE: 2023-10-04_04-28-11

Refinement model description

Number of restraints - 149, number of constraints - 0.

Details:

```
1. Restrained distances
 C7-H7 = C3-H3 = C4-H4 = C6-H6
 1.085 with sigma of 0.02
 N1-H1b = N2-H2b = N2-H2a = N1-H1a
 1.04 with sigma of 0.02
 C8-H8c ≈ C8-H8d ≈ C8-H8a ≈ C8-H8e ≈ C8-H8b ≈ C8-H8f
 with sigma of 0.01
 H8a-H8b ≈ H8a-H8c ≈ H8b-H8c
 with sigma of 0.04
 H8a-H8b ≈ H8a-H8c ≈ H8b-H8c
 with sigma of 0.04
 H8d-H8e ≈ H8d-H8f ≈ H8e-H8f
 with sigma of 0.04
2. Uiso/Uaniso restraints and constraints
H8b ≈ H8f ≈ H8c ≈ H8d ≈ H8a ≈ H8e: within 2A with sigma of
0.04 and sigma for terminal atoms of 0.08 within 2A
Uanis(H8e) ≈ Ueq, Uanis(H8b) ≈ Ueq, Uanis(H8f) ≈ Ueq, Uanis(H8c)
≈ Ueq, Uanis(H8a) ≈ Ueq, Uanis(H8d) ≈ Ueq: with sigma of 0.01 and
sigma for terminal atoms of 0.02
Uanis(H1b) ≈ Ueq, Uanis(H1a) ≈ Ueq, Uanis(H2b) ≈ Ueq, Uanis(H2a)
≈ Ueq: with sigma of 0.01 and sigma for terminal atoms of 0.02
Uanis(H7) ≈ Ueq, Uanis(H3) ≈ Ueq, Uanis(H4) ≈ Ueq, Uanis(H6) ≈
Ueq: with sigma of 0.01 and sigma for terminal atoms of 0.02
3. Rigid body (RIGU) restrains
 C7, H7
 with sigma for 1-2 distances of 0.004 and sigma for 1-3 distances of 0.004
 C6, H6
 with sigma for 1-2 distances of 0.004 and sigma for 1-3 distances of 0.004
 C3, H3
 with sigma for 1-2 distances of 0.004 and sigma for 1-3 distances of 0.004
 C4, H4
 with sigma for 1-2 distances of 0.004 and sigma for 1-3 distances of 0.004
 N2, H2a
 with sigma for 1-2 distances of 0.004 and sigma for 1-3 distances of 0.004
 N2, H2b
 with sigma for 1-2 distances of 0.004 and sigma for 1-3 distances of 0.004
 N1, H1a
 with sigma for 1-2 distances of 0.004 and sigma for 1-3 distances of 0.004
 N1, H1b
 with sigma for 1-2 distances of 0.004 and sigma for 1-3 distances of 0.004
4. Others
 Fixed Sof: H8a(0.5) H8b(0.5) H8c(0.5) H8d(0.5) H8e(0.5) H8f(0.5)
```

This report has been created with Olex2, compiled on 2023.08.24 svn.re1ec1418 for OlexSys. Please let us know if there are any errors or if you would like to have additional features.

4. Additional references for the Supplementary Information

S1 Schäffer, A, Ph.D. Thesis, Univ. of Münster, **1984** (via private communication from Prof. Dr. B. Krebs).

S2 Dolomanov, O.V., Bourhis, L.J., Gildea, R.J, Howard, J.A.K. & Puschmann, H. *J. Appl. Cryst.* **2009**, *42*, 339-341.

S3 Sheldrick, G.M. Acta Cryst. **2008**, A*64*, 112-122.

S4 Bourhis, L.J., Dolomanov, O.V., Gildea, R.J., Howard, J.A.K., Puschmann, H. *Acta Cryst.* **2015**, A*71*, 59-75.

S5 Kleemiss, F.; Dolomanov, O.V.; Bodensteiner, M.; Peyerimhoff, N.; Midgley, L.; Bourhis, L.J.; Genoni, A.; Malaspina, L.A.; Jayatilaka, D.; Spencer, J.L.; White, F.; Grundkotter-Stock, B.; Steinhauer, S.; Lentz, D.; Puschmann, H.; Grabowsky, S. Accurate crystal structures and chemical properties from NoSpherA2. *Chem. Sci.* **2021**, *12*, 1675−1692.
